# A novel cochlear measurement that predicts inner-ear malformation

**DOI:** 10.1038/s41598-021-86741-x

**Published:** 2021-04-01

**Authors:** Tawfiq Khurayzi, Fida Almuhawas, Abdulrahman Alsanosi, Yassin Abdelsamad, Úna Doyle, Anandhan Dhanasingh

**Affiliations:** 1 King Abdullah Ear Specialist Center (KAESC), College of Medicine, King Saud University, Riyadh, 11411 Saudi Arabia; 2 Research Department, MED-EL GmbH, Riyadh, Saudi Arabia; 3 Research and Development Department, MED-EL GmbH, Innsbruck, Austria; 4 Department of Translational Neurosciences, Faculty of Medicine and Health Sciences, University of Antwerp, Antwerp, Belgium; 5 King Fahad Central Hospital, Ministry of Health, Jizan, 82666 Saudi Arabia

**Keywords:** Anatomy, Health care, Medical research

## Abstract

The A-value used in cochlear duct length (CDL) estimation does not take malformed cochleae into consideration. The objective was to determine the A-value reported in the literature, to assess the accuracy of the A-value measurement and to evaluate a novel cochlear measurement in distinguishing malformed cochlea. High resolution Computer Tomography images in the oblique coronal plane/cochlear view of 74 human temporal bones were analyzed. The A-value and novel C-value measurement were evaluated as predictors of inner ear malformation type. The proximity of the facial nerve to the basal turn was evaluated subjectively. 26 publications report on the A-value; but they do not distinguish normal *vs.* malformed cochleae. The A-values of the normal cochleae compared to the cochleae with cochlear hypoplasia, incomplete partition (IP) type I, -type II, and -type III were significantly different. The A-value does not predict the C-value. The C-values of the normal cochleae compared to the cochleae with IP type I and IP type III were significantly different. The proximity of the facial nerve to the basal turn did not relate to the type of malformation. The A-value is different in normal *vs.* malformed cochleae. The novel C-value could be used to predict malformed anatomy, although it does not distinguish all malformation types.

## Introduction

The measurement of the cochlear duct length (CDL) was first reported by direct measurement in 1884 and many years later, the CDL was determined by indirect measurement and graphic reconstruction^1^; following which Escudé et al. introduced the ‘A-value’ measurement of the cochlea^2^ and proposed mathematical equations to estimate the CDL along the outer wall using the A-value. Alexiades et al.^4^. later described how the A-value could reliably be used to estimate the CDL along the basilar membrane, after modifying the equations derived by Escudé; demonstrating results that were in agreement with the outcomes of Hardy’s histological study^3,4^.

In the oblique-coronal plane, the basal turn of the cochlea can be viewed fully, along with the round window (RW) entrance and the three semi-circular canals of the vestibular organ. This has been termed the ‘Cochlear View’ by Xu et al.^5^. The ‘cochlear view’ is the best view in which to measure the A-value. The position of the facial nerve in relation to the electrode array can also be identified accurately in the ‘cochlear view’.

In the ‘cochlear view’ we have experienced that the curvature of the cochlear turn of the cochlea is highly variable^6^. If the beginning of the second turn of the cochlea is far away from the RW, the curvature of the second turn of the cochlea becomes very tight, affecting the insertion angle considerably. Likewise, Escudé et al. had suggested that the cochlear size influences greatly the final insertion depth of the electrode^2^. While the A-value has been recognized as a reliable measurement for CDL estimation for cochlear implantation, it does not account for variability in the curvature of the second turn of the cochlea. Furthermore, the A-value can only be applied to inner ears with a normal anatomy^7^, because malformed cochlea generally do not possess two and a half turns like the normal cochlea; the cochlear duct is typically shorter^8^.

This is of importance because up to 20% of the population with a sensorineural hearing loss have some degree of inner ear malformation ^9^, with enlarged vestibular aqueduct (EVA), incomplete partition (IP) types I, II and III, cochlear hypoplasia (CH), common cavity (CC), and cochlear aplasia (CA) being the most common ^7,10^. Therefore, applying the A-value measurement and CDL estimation under circumstances where the cochleae have a malformation of any kind be ill advised. In such cases, it is necessary to consider the anatomy of the cochlea and approach the estimation of the CDL individually.

Therefore, this study set out to determine the reliance on the A-value measurement in both normal and malformed cochleae. We hypothesized that the A-value is different in normal versus malformed cochleae. We also sought an alternative measurement that could be used as a landmark to distinguish between normal and malformed cochlea.

## Methods

- To determine the present-day reliance on the A-value measurement, a literature search was performed.
- To assess the accuracy of the A-value measurement, in normal and malformed cochleae, image analyses of pre-operative high resolution Computer Tomography (HRCT) image datasets in the ‘cochlear view’ of anonymous human temporal bones shared by multiple clinics for educational purposes from the year 2011 to 2020 were analyzed retrospectively.
- As an alternative landmark, the gap between the inner wall at the beginning of the cochlea to the outer wall at the beginning of the second turn of the cochlea was measured. This novel anatomical indicator was termed the C-value measurement.

### Literature summary

A literature search of the key words “Cochlear duct length measurement” was performed using PubMed as the search engine. All studies that reported on the A-value taking in cadaveric human temporal bones and live human subjects were included in the analyses.

### Image analyses

Seventy-four preoperative HRCT images were analyzed retrospectively using 3D slicer, version 4.10.2, freeware (https://www.slicer.org/).

The A-value was determined in the oblique coronal plane starting at the entrance of the round window and passing through the mid-modiolar section to the opposite side of the lateral wall in ‘cochlear view’ as described previously by Escudé et al.^2^.

The C-value was determined in the oblique coronal plane/cochlear view and along the ‘A’ value line by measuring the gap between the inner wall at the beginning of the cochlea to the outer wall at the beginning of the second turn of the cochlea.

The proximity of the facial nerve to the basal turn of the cochlea was evaluated subjectively in all 74 image datasets.

Three-dimensional (3D) segmentation of the complete inner-ear was performed using 3D slicer as described previously by Dhanasingh et al. ^7,11^.

### Statistical analyses

The A- and C-values of the normal and malformed cochleae were compared using two sample *t-*tests with unequal variance in Microsoft Excel for Office 365 (Version 2020).

Regression estimates between the A- and C-values of each type of cochlea (normal vs. malformation) were determined using the data analysis tool in Microsoft Excel. A *p*-value < 0.05 was considered statistically significant.

## Results

### Literature summary

**Figure 1:**
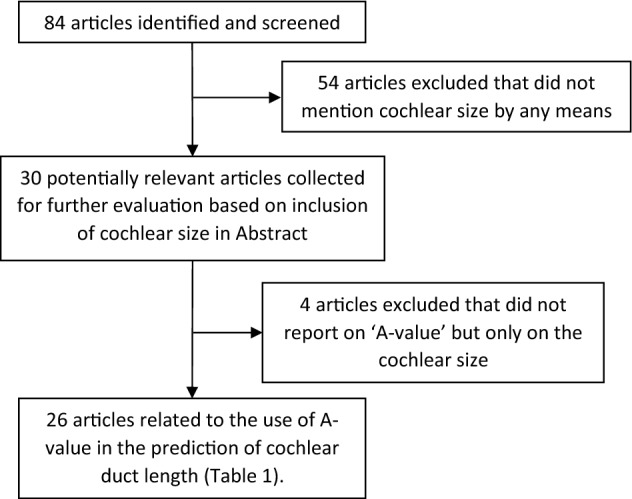
Identification of studies reporting on the A-value of cochlea.

Eighty-four articles in total were retrieved from the literature search and upon reading the abstract thoroughly and reviewing the articles briefly for presence of the A-value, twenty-six articles were identified that satisfied the inclusion criteria (Fig. 1). The literature search results are shown in Table 1^2,4,12–35^. From these twenty-six reports, a total of three-thousand-three-hundred and thirty-three cochleae were analyzed for the A-value. The A-value from this literature review ranged from a minimum of 7.9 mm to a maximum of 10.2 mm with an average value of 9.13 mm. None of the articles within the inclusion criteria reported on the A-value in malformed cochleae.

**Table 1:** Literature search of studies pertaining to A-value measurement.

No	Study	N	Mean A-value	A-value range
1	Ordonez et al.^12^	5	10.45 ± 0.18	–
2	Khurayzi et al.^13^	256	8.45	7.5–9.4
3	Nateghifard et al.^14^	10	8.89 ± 0.3	–
4	Kuthubutheen et al.^15^	55	8.91 ± 0.37	–
5	Nash et al.^16^	40	9.1 ± 0.49	–
6	Zahara et al.^17^	36	8.75 ± 0.31	–
7	Hong et al.^18^	120	8.55 ± 0.31	–
8	Stefanescu et al.^19^	23	9.14 ± 0.415	–
9	Schurzig et al.^20^	10	9.61 ± 0.54	–
10	Iyaniwura et al.^21^	20	9.05	8.4–9.7
11	Grover et al.^22^	124	8.45	7.7–9.2
12	An et al.^23^	26	9.75	9.0–10.5
13	Liu et al.^24^	102	8.85	8.1–9.6
14	Rivas et al.^25^	275	9.22 ± 0.44	8.0–10.3
15	Deep et al.^4^	40	9.5	8.5–10.5
16	Thong et al.^26^	314	9.2	8.1–10.3
17	Mosnier et al.^27^	8	9.3 ± 0.44	–
18	Meng et al.^28^	310	9.3	8.1–10.5
19	Franke-Trieger et al.^29^	10	9.0	8.3–9.67
20	Van der Marel et al.^30^	671	9.1	7.3–10.9
21	Avci et al.^31^	16	9.5	8.8–10.1
22	Pelliccia et al.^32^	482	9.3	7.14–11.4
23	Erixon et al.^33^	325	9.1	8.3–9.9
24	Martinez-Monedero et al.^34^	104	8.5	6.8–10.3
25	Stakovskaya et al.^35^	9	9.1	7.3–10.9
26	Escudé et al.^2^	42	9.23 ± 0.53	7.9–10.8
		**3433**	**9.13**	**7.9–10.2**

### Data analyses

**Figure 2:**
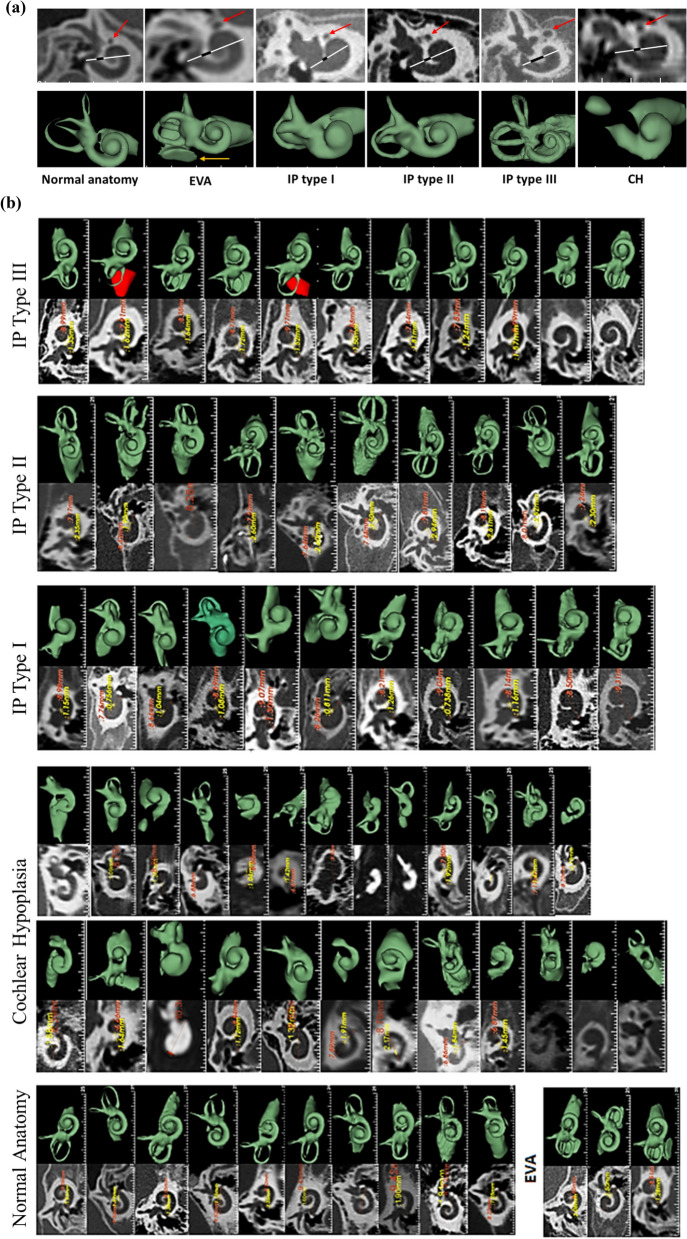
Two- and three-dimensional images of different cochlear anatomies, displayed in the oblique coronal plane/cochlear view, showing the A-value (white line), C-value (black line) and the facial nerve (red arrow) (**a**); and all seventy-four HRCT images of the cochlear samples used in the analyses along with the corresponding 3D images (**b**).

Of the seventy-four cochleae investigated there were: 10 normal, 3 with EVA, 25 with CH, 15 with IP type I, 11 with IP type II, and 10 with IP type III. HRCT images with the A-value and C-value, and corresponding 3D segmented images covering all the anatomical types of the inner-ear in cochlear view are shown in Fig. 2a. Images of all the seventy-four cochlear samples used are provided in Fig. 2b.

**Figure 3:**
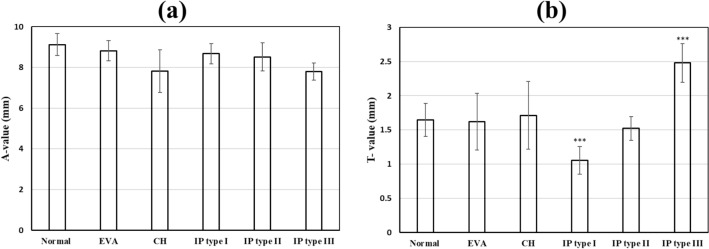
Histogram showing the mean ± standard deviation of A-values (**a**), and C-values (**b**), of normal versus malformed cochleae captured from CT images used in this study. Asterisks indicate statistical significance where *p < 0.05; **p < 0.01; and ***p < 0.001.

The A-values and the C-Values (mean ± standard deviation) for each group are shown in Fig. 3a,b, respectively. The A-values of the normal cochleae compared to the cochleae with CH (*p* < 0.001), IP type I (*p* = 0.049), IP type II (*p* = 0.038), and IP type III (*p* < 0.001) were significantly different. However, the A-values of the normal cochleae were not significantly different to the cochleae with EVA (*p* < 0.430). The C-values of the normal cochleae compared to the cochleae with IP type I (*p* < 0.001) and IP type III (*p* < 0.001) were significantly different, but there were no significant differences between the C-value and the other malformation types.

**Figure 4:**
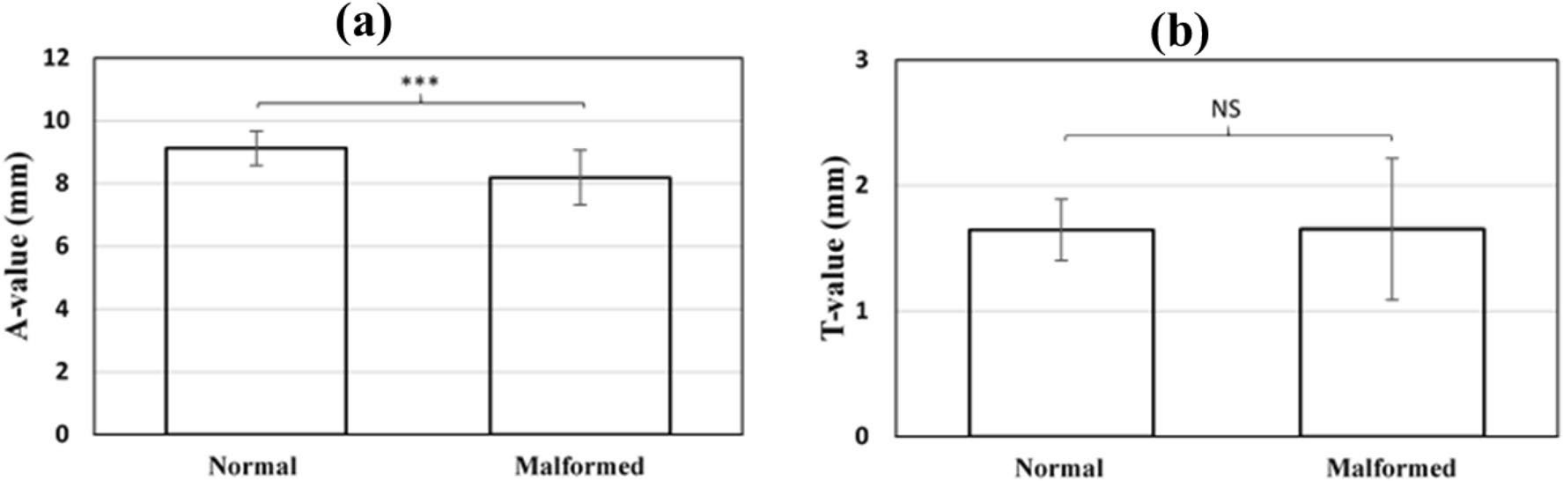
Comparison of mean ± standard deviation of A- and C-values of normal cochleae versus malformed cochleae (combined values of all the malformation types) captured from CT images used in this study. Asterisks indicate statistical significance where *** = *p* < 0.001 and *NS* not significant.

The A-values of the normal cochleae were significantly different to the combined A-values of the cochleae with a malformation (*p* < 0.001) (Fig. 4a). The C-values of the normal cochleae were not significantly different to the combined C-values of the cochleae with a malformation (Fig. 4b).

Table 2 shows the exact A- and C-values (mean ± standard deviation) for the normal cochleae and each type of malformation, the *p*-values of the differences as determined by *t*-test between the A- and C-values, and the *p*-values of the regression analyses.

**Table 2:** Statistical significance of A and C values of different anatomical types of cochleae.

	A-value (mean ± SD)	C-value (mean ± SD)	*p*-value *t*-test A vs C	*p*-value regression A vs C
Normal	9.12 ± 0.54	1.65 ± 0.24	< .001	0.693
EVA	8.82 ± 0.50	1.62 ± 0.41	< .001	0.863
CH	7.82 ± 1.04	1.71 ± 0.49	< .001	0.104
IP type I	8.67 ± 0.49	1.05 ± 0.20	< .001	0.262
IP type II	8.51 ± 0.69	1.52 ± 0.18	< .001	0.456
IP type III	7.80 ± 0.42	2.48 ± 0.28	< .001	0.562

**Figure 5:**
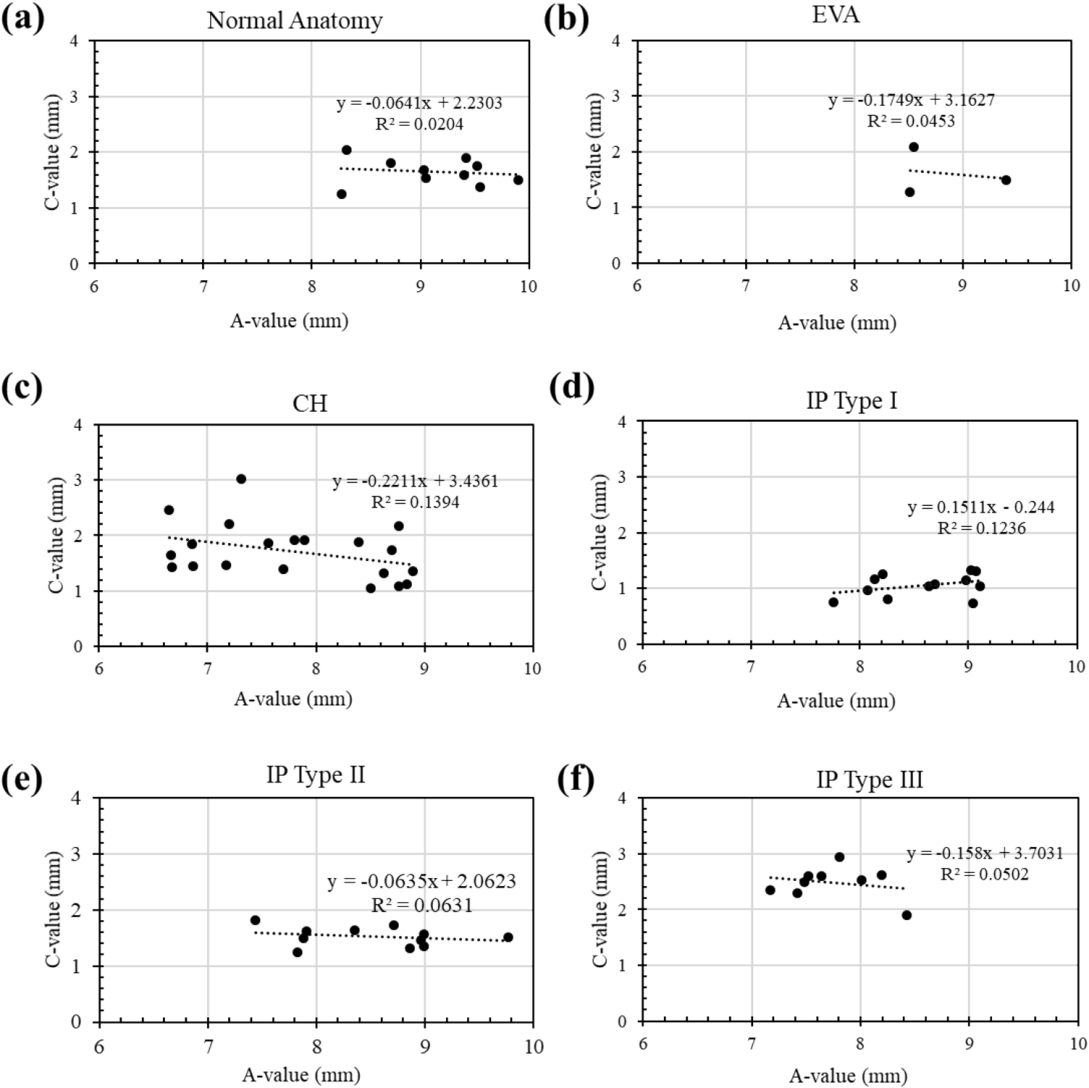
Plots of regression of A- versus C- value of normal anatomy (**a**), EVA (**b**), CH (**c**), IP type I (**d**), IP type II (**e**), and IP type III (**f**) captured from CT images used in this study.

The A-value of the normal cochleae (Fig. 5a), EVA (Fig. 5b), CH (Fig. 5c), IP type I (Fig. 5d), IP type II (Fig. 5e), or IP type III (Fig. 5f) were not significant predictors of the corresponding C-value.

The proximity of the facial nerve to the cochlear basal turn of the cochlea at around 270° of angular depth, was evaluated subjectively in all 74 samples and it was determined that the proximity was highly variable, regardless of the anatomical type (Fig. 2b).

## Discussion

The data collected shows that while the A-value measurement has been reported in the literature and is used to determine the CDL in clinical practice, the A-value measurement varies greatly. A-value measurements for CDL prediction apply only to normal cochleae as the mathematical equations to estimate CDL are derived taking two-and half turns of the fully formed cochlea into account. However, the malformed cochleae have defects in the full development of the cochlear turns making it difficult to apply the mathematical equations to estimate the available length of cochlear lumen for electrode placement. The C-value measurement is a novel dimension of the cochlea presented herein and it distinguishes certain types of malformed cochlea from normal cochlea.

The introduction and use of the A-value measurement to the field of cochlear implantation means that the otolaryngologist can quickly determine pre-operatively, via CDL estimation, the length of the electrode that is best suited for the cochlea. Twenty-six papers were published on the A-value measurement since 2006 of which sixteen papers were published in the last two years showing the importance given to the A-value in present-day cochlear implant therapy. However, none of the papers that reported on the use of the A-value distinguished between normal and malformed cochleae.

In the early days of cochlear implantation, patients with a malformation were initially excluded from cochlear implantation. However, these days a high percentage of patients with inner ear malformations are undergoing surgery successfully^36^. Despite significant benefits in sound detection, some risks and surgical challenges for these patients still exist^37–41^, e.g. facial nerve stimulation is one of the most common complications of CI surgery and cochlear malformation has been associated with a higher incidence of facial nerve stimulation^42^. It is thought that the location of the labyrinthine segment of the facial nerve in relation to the superior segment of the basal turn of the cochlea leads to the facial nerve stimulation^37,43^. Therefore, to avoid such risks a proper understanding of the topographic anatomy of the cochlea is essential. In particular, the use of one plane, the oblique coronal plane, or ‘cochlear view’ is advised. We have shown herein that determining the A-value in the ‘cochlear view’ the mean A-value was 9.12 ± 0.89 in normal cochleae (n = 10). This value is very much in-line with the findings in the literature that showed an average A-value of 9.13 mm measured from a sample number of 3433. However, the A-value was significantly lower in malformed cochleae (8.18 ± 0.86). Specifically, the A-value was significantly lower in CH (*p* < 0.001) and IP type III (*p* < 0.001); and it was marginally lower in IP type I (*p* = 0.05), -II (*p* = 0.04), and EVAS (*p* = 0.5) cochleae, compared to the normal cochleae (Fig. 3a). This indicates that the A-value is not an appropriate measurement in malformed cochleae.

If one undertakes the A-value measurement without considering the apical anatomy of the cochlea, the A-value measurement will yield a CDL with a false measurement in malformed cochleae. In clinical practice this leads to selecting the wrong electrode. For example, using a longer than necessary electrode, which increases the probability of cochlear trauma, or partial insertion. Recent reports from our clinic, indicate that electrode tip fold-over is a consequence if the full insertion of a long electrode is attempted in a malformed cochlea with a cystic apex^44^.

The C-value is a novel cochlear dimension introduced in this study. The A-value is significantly different to the C-value. The regression analyses showed that the A-value does not predict the C-value of the individual malformation types. The C-value measurement of normal cochleae was significantly different to the C-value measurement of IP type I (p < 0.001) and IP type III (p < 0.001) cochleae, but not significantly different to the other malformation types (Fig. 3b). Thus, the C-value could be used as a quantitative positive predictor of the malformation types IP type I and IP type III, but it does not completely distinguish all malformation types. Therefore, we recommend the 3D segmentation of the complete inner ear structures to determine malformation types and to choose the appropriate length electrode. It remains to be seen, were we not constrained by the number of HRCT images available, if the method could predict with greater accuracy the presence of malformed cochleae or different types of malformation.

Based on the HRCT images that we had available to us, we would predict that the maximum electrode coverage would be 360° for an IP type I; 450° for an IP type II, and 360° for an IP type III malformed cochlea. The CH type malformation shows great variation in the cochlear turns available and, as the data indicated, they differ significantly to the normal cochleae in terms of the A-value and on the number of turns available. However, based on the CH samples presented in this study, the ideal electrode coverage for malformation types would vary from 180° to 360°. The number of EVA malformation samples presented in this study was too small (n = 3) to enable prediction. However, EVA is thought to have the appearance of a normal cochlear part with the vestibular aqueduct being enlarged. Therefore, the electrode choice for normal anatomy cochlea could be applied to EVA, if no defect in the cochlear portion was detected.

The oblique coronal plane/cochlear view is a ‘one-stop shop’ view in which the A-value, C-value and even the proximity of the facial nerve to the basal turn of the cochlea can be measured. Our previously published data showed that patients who experience postoperative facial nerve stimulation have a significantly lower distance and bone density between the upper basal turn of the cochlea and the labyrinthine segment of the facial nerve^45^. However, subjective evaluation of the proximity of the facial nerve to the basal turn in the present study did not appear to have any relationship to the type of malformation.

## Conclusion

In conclusion, the scientific evidence is in favor of the A-value measurement in modern cochlear implant therapy. The novel C-value measurement could prospectively be used to predict malformed anatomy, although both the A-value and the C-value do not distinguish all malformation types. Oblique coronal view/cochlear view is a complete solution in which to measure the cochlear parameters and to visualize the proximity of the facial nerve to the basal turn of the cochlea. 3D segmentation of the complete inner-ear especially in malformed cochlear anatomies should be considered as a routine in pre-operative image analysis and in our experience we find it highly useful in understanding the anatomy of the malformed cochleae.

## Data Availability

All data generated or analyzed during this study are included in this published article.
